# Telehealth pulmonary rehabilitation enhances functional capacity and quality of life in veterans with chronic obstructive pulmonary disease: a 12-week single-arm intervention study

**DOI:** 10.1038/s41598-024-84518-6

**Published:** 2025-11-28

**Authors:** Abderrahman Ouattas, Mehrnaz Azarian, Mon S. Bryant, Christina K. Nguyen, Naima Rodriguez, Ilse Paulette Torres Ruiz, Amir Sharafkhaneh, Bijan Najafi

**Affiliations:** 1 Present Address: Department of Surgery, Center for Advanced Surgical and Interventional Technology (CASIT), David Geffen School of Medicine, University of California, Los Angeles, 700 Westwood Plaza, Suite 2207, Los Angeles, CA 90065 USA; 2 Telehealth Pulmonary & Cardiac Rehabilitation, Michael E. DeBakey VA Medical Center (MDVAMC), Baylor College of Medicine, Houston, TX 77030 USA; 3 Department of Medicine, Section of Pulmonary, Critical Care and Sleep Medicine, Baylor College of Medicine, Houston, TX USA; 4 Interdisciplinary Consortium on Advanced Motion Performance (iCAMP), Michael E. DeBakey Department of Surgery, Baylor College of Medicine, One Baylor Plaza, MS: BCM 390, Houston, TX 77030 USA

**Keywords:** Rehabilitation, Randomized controlled trials

## Abstract

Traditional in-person pulmonary rehabilitation protocols enhance respiratory health and functional capacity in Chronic Obstructive Pulmonary Disease (COPD) patients but face scalability issues due to high dropout rates and poor acceptability. Telerehabilitation offers a promising alternative. This 12-week single-arm intervention study evaluated the acceptability, feasibility, and effectiveness of a telehealth pulmonary rehabilitation (TPR) program for improving functional capacity in Veterans with COPD. Of the 51 participants, 44 (86% retention) completed the TPR program via the secure online VA Video Connect platform, which included weekly 120-min sessions. At the 12-week mark, compared to baseline, significant improvements were observed in functional capacity (41.3 m = 15.7% increase in the 6-min walk distance), functional mobility (1.2 s = 9.94% faster in Timed Up and Go), lower-body strength (1.2 s = 8.98% quicker in 5-Times Sit to Stand), and COPD-affected quality of life (27.9% improvement in St. George’s Respiratory Questionnaire and 42.7% in COPD Assessment Test). The TPR intervention, with its low dropout rate, proves to be an accessible and effective method for enhancing functional capacity and mobility among Veterans with COPD, potentially benefiting veterans and others with chronic respiratory conditions.

## Introduction

Chronic obstructive pulmonary disease (COPD) is a progressive and fatal respiratory disorder that not only targets the pulmonary system, but also leads to a debilitation in functional performance including muscle weakness^1^, fatigue^2^, higher frailty^3^, and increased risk of falls^4^. Globally, around 400 million people are affected by COPD^5^. Amongst those, about 15.7 million COPD patients are living in the US^6^, and COPD is being reported as the third leading cause of death in 2018^7,8^. This rate is even higher in veterans (5–43% in veterans versus 5–15% in the adult US population)^9–11^, and it might be attributed to the strong association between combat experience and health risk behaviors including smoking^12^ or exposure to chemical agents^13–15^. The Department of Veterans Affairs (VA) provides annual care for approximately 500,000 COPD cases^16^, making it one of the most common discharge diagnoses from VA hospitals^17^.

Extra-pulmonary consequences of COPD negatively affect whole-body biomechanics (e.g., gait, balance, range of motion, etc.)^18^, decrease ambulatory and physical activity^19–21^, and impair overall quality of life^22^. Pulmonary rehabilitation (PR) is an evidence-based, comprehensive intervention that melds exercise training with educational sessions for patients with chronic respiratory diseases^23^. PR reduces breathlessness and improves physical capabilities and overall well-being in patients with COPD including veterans^24–26^. However, low patient retention and adherence to PR programs often occurs due to patient frailty, transportation issues, and healthcare access problems^27–29^ and can hinder its effectiveness and cause recurrence of the symptoms^30^.

Tele-rehabilitation, utilizing information and communication technologies, offers a remote alternative to traditional center-based programs^31^. PR delivered at home may enhance the integration of exercise into daily life,^32^ potentially providing long-lasting benefits.^33,34^. The Department of Veterans Affairs has adopted VA Video Connect (VVC), enabling clinicians to offer tele-rehabilitation directly to veterans in their homes, addressing previous barriers to adherence and improving access for those in rural areas^35^.

Data on the efficacy of using telehealth pulmonary rehabilitation to improve functional capacity, strength, and mobility in veterans with COPD is limited. Our study aimed to assess the effectiveness of a 12-week telehealth pulmonary rehabilitation (TPR) program on functional capacity, as measured by the distance covered during six-minute walk test (6MWT). The 6MWT evaluates functional capacity by assessing the integrated response of cardiovascular, pulmonary, circulatory, neuromuscular, and musculoskeletal systems and has been validated in COPD patients^36^. We hypothesized that functional capacity, as measured by the 6MWT, would improve following the 12-week TPR intervention. Secondary hypotheses included a high retention rate (with less than 20% dropout) and positive effects on functional mobility (measured through the Timed Up & Go (TUG) test), lower-body strength (measured through the Five Times Sit-to-Stand (STS) test), and quality of life (measured through the St. George’s Respiratory Questionnaire (SGRQ) and COPD Assessment Test (CAT)).

## Methods

### Study design

This is a 12-week single-arm cohort intervention study aimed at investigating the effectiveness, acceptability, and feasibility of an in-home, interactive, supervised telehealth pulmonary rehabilitation (TPR) program, delivered through the VA Video Connect (VVC). The study adhered to the CONSORT-EHEALTH (Consolidated Standards of Reporting Trials of Electronic and Mobile Health Applications and online TeleHealth) checklist to meet the reporting standards for interventional studies^37^. All eligible veterans with COPD referred to TPR program at the Michael E. DeBakey Veterans Affairs Medical Center (MDVAMC) between July 3rd, 2018, and March 6th, 2020, were invited to participate.

### Study population

All veterans (aged 18 or older) with clinically diagnosed Chronic Obstructive Pulmonary Disease (COPD) living within the Greater Houston metropolitan area, Texas, USA—including those in rural and highly rural areas—referred to and accepted for the Telehealth Pulmonary Rehabilitation (TPR) program at the MDVAMC were invited to participate in this study. Participants were excluded if they (1) had a surgery that affects the participants’ mobility, and (2) had any neurological disease or impairment that limits their ability to walk (e.g., Parkinson’s disease, multiple sclerosis), (3) were unlikely to fully comply with the protocol (e.g., long distance travel to attend any in person visit, at baseline and follow-up), (4) were unwilling to provide informed consent.

The intervention study and procedures involved, including the risks, were explained and discussed with all participants prior to their involvement in the research study. All procedures were explained in detail to each participant over the phone before their visit and reviewed a second time during the initial visit in a quiet and private room at MDVAMC before signing the informed consent. Informed consent was obtained, and research procedures were conducted in accordance with the Declaration of Helsinki. The intervention study was approved by the Institutional Review Board of both MDVAMC and the Baylor College of Medicine (IRB# H-40765).

### Protocol

#### Telehealth pulmonary rehabilitation (TPR) Intervention

**Fig. 1:**
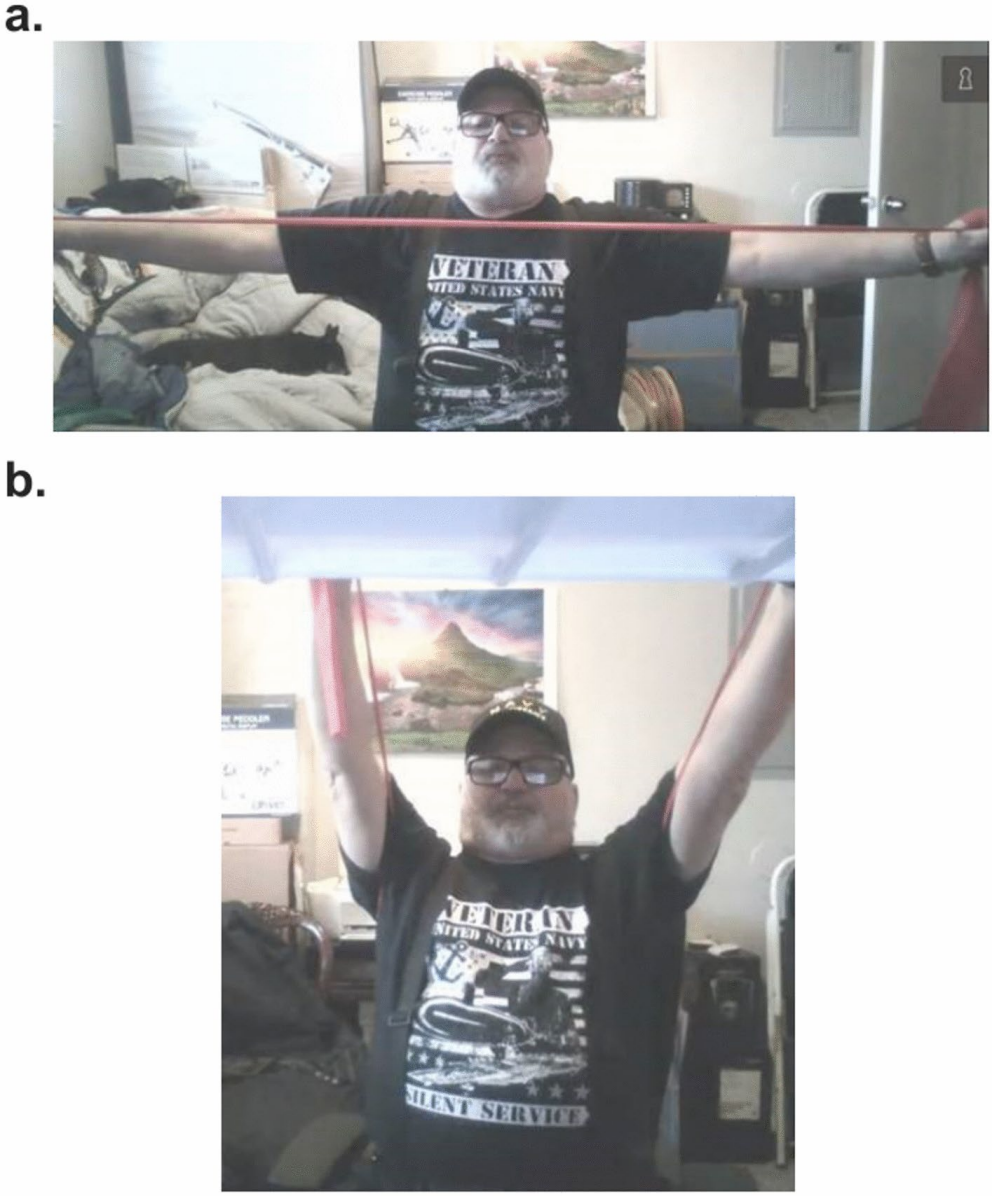
A telehealth demo session of the Telehealth Pulmonary Rehabilitation (TPR) performed using the VA Video Connect (VVC) developed by the Veteran Health Administration (VHA). (**A**) Participant performing upper body strengthening exercises. (**B**) Participant performing lower body strengthening exercises.

The secure online VA Video Connect (VVC), developed by the Veteran Health Administration (VHA) facilitated the delivery of an in-home interactive, supervised TPR program (Fig. 1). The intervention has been previously described in detail^35^. 44 veterans with COPD participated in a 12-week program once a week. Each session, lasting about 120 min, was conducted by both a licensed physical therapist and a respiratory therapist. Participants were provided with an exercise pictorial booklet, an exercise compact disc (audio and video), pulse oximeter, blood pressure monitor, and were trained to self-measure blood pressure, heart rate, SpO2, respiratory rate and level of exertion during the exercise session. Sessions began with clinical self-measures (i.e., resting blood pressure, heart rate, and blood oxygen saturation), and continued with the therapists monitored clinical symptoms (e.g., fatigue, dyspnea) throughout the entirety of the session. Each session included stretching and warm-up exercises, followed by progressive (increase in volume and load based on patient progress and comorbidity; sets, repetitions, and level of difficulty) strengthening, aerobic, balance, and flexibility exercises. Strengthening, balance, and flexibility exercises consisted of chair squats, chair stands, knee marching, bridging, single limb stances, and lunges. Aerobic exercises consisted of daily walking and a provided arm/pedal ergometer. Additionally, the respiratory therapist provided muscle training and breathing exercises such as huff coughing, diaphragm breathing, and pursed-lip breathing. Dropout rates were quantified by the number of participants that failed to complete the 12-week TPR due to any reason other than the mandatory discontinuation of research because of the COVID-19 pandemic.

#### Outcomes measure

Outcome assessments were conducted at baseline and again at 12 weeks during in-person visits at the Michael E. DeBakey Veterans Affairs Medical Center in Houston, TX, USA. The primary outcome measured changes over the 12-week period compared to baseline in functional capacity, as quantified by the Six-Minute Walk Test (6MWT). Secondary outcomes included the evaluation of changes in lower-body strength, functional mobility, and quality of life at the 12-week mark relative to baseline. These were quantified using the Five Times Sit-to-Stand Test (5XSTS) for lower-body strength, the Timed Up and Go Test (TUG) for functional mobility, and the St. George’s Respiratory Questionnaire (SGRQ), and the COPD Assessment Test™ (CAT) for quality of life. A rehabilitation program nurse completed a clinical check-up for each patient upon their arrival to assure safety. Demographics and clinical characteristics were collected at each visit.

#### Six-minute walk test (6MWT)

A 50-m hallway was thoroughly inspected in advance to ensure it was flat, hazard-free, and suitable for an unobstructed walk. Visible markers were placed every 10 m. Participants were instructed to cover as much distance as possible while walking for six minutes. Upon reaching the six-minute time point, the participant was instructed to stop; the cessation point was marked, and the distance from the nearest 10-m mark was calculated using a tape measure and added to the total covered distance. The 6MWT measures functional capacity by assessing the global and combined performance of cardiovascular, pulmonary, circulation, neuromuscular and musculoskeletal responses, and was validated in patients with COPD^38–40^.

#### Timed up and go test (TUG)

Participants were asked to rise from a standard height chair (seat height 46 cm) without support, walk 3 m, and return to a seated position in the same chair. Time of completion was recorded in seconds as the outcome measure. The TUG measures functional mobility, dynamic balance and risk of falling and could predict morbidity and mortality in several clinical populations, including patients with COPD^41–47^.

#### Five times sit-to-stand test (5XSTS)

While a standard height armless chair was stabilized (seat height 46 cm), participants were asked to rise all the way and sit down five times with their arms crossed across their chests. Time from start to completion of all five sit-to-stands was recorded in seconds, and collected as an outcome measure. The 5XSTS measures functional lower limb strength and balance and it has been validated in several clinical populations, including patients with COPD^48^.

#### Quality of life (QoL)

The St. George’s Respiratory Questionnaire (SGRQ), and the COPD Assessment Test™ (CAT), were used to assess the impact of COPD on overall health, wellbeing, and daily life. Higher scores indicate worse quality of life affected by COPD^49,50^.

### Sample size estimation

A priori sample size estimation was performed using G*Power software (version 3, Düsseldorf, Germany). Tsai et al. demonstrated in their randomized control trial the effect of home-based tele-rehabilitation and reported a medium time effect size for improving the six-minute walk distance in patients with COPD (Cohen effect sizes d of 0.53)^51^. We recruited 50 participants assuming an attrition rate of 10% (n = 10), a total sample size of 40 participants, with an anticipated medium time effect size of Cohen’s d = 0.53 and alpha of 0.05, yields a power of 0.90.

### Statistical analyses

All data were assessed for normality using Shapiro–Wilk test, visual inspection of the histograms, Q-Q plots, and box plots. To assess the effectiveness of our telehealth pulmonary rehabilitation intervention, a two-tailed paired sample t-test was used to assess the differences in 6MWT, 5XSTS, TUG, and QoL scores between the baseline visits, prior to the involvement with the intervention, and 12-week follow up visits, post completion of the intervention. Additional sub-analyses were completed to assess the differences in demographic and clinical characteristics between participants who completed all milestones of the study compared to the remaining participants that consented using generalized linear models to account for non-normal distributions. The outcome measures were blindly measured by the author (AO) that performed data and statistical analyses. Adobe Illustrator (Adobe Inc., Mountain View, CA, USA) and custom MATLAB codes (MathWorks, R2023a, Natick, MA, USA) were used for data visualization, and SPSS (IBM, v.29, Armonk, NY, USA) was used for statistical analyses. The significance level was set at α < 0.05, and effect sizes were calculated using Cohen’s d, interpreted as follows; small (d = 0.2), medium (d = 0.5), and large (d = 0.8)^52^.

## Results

**Fig. 2:**
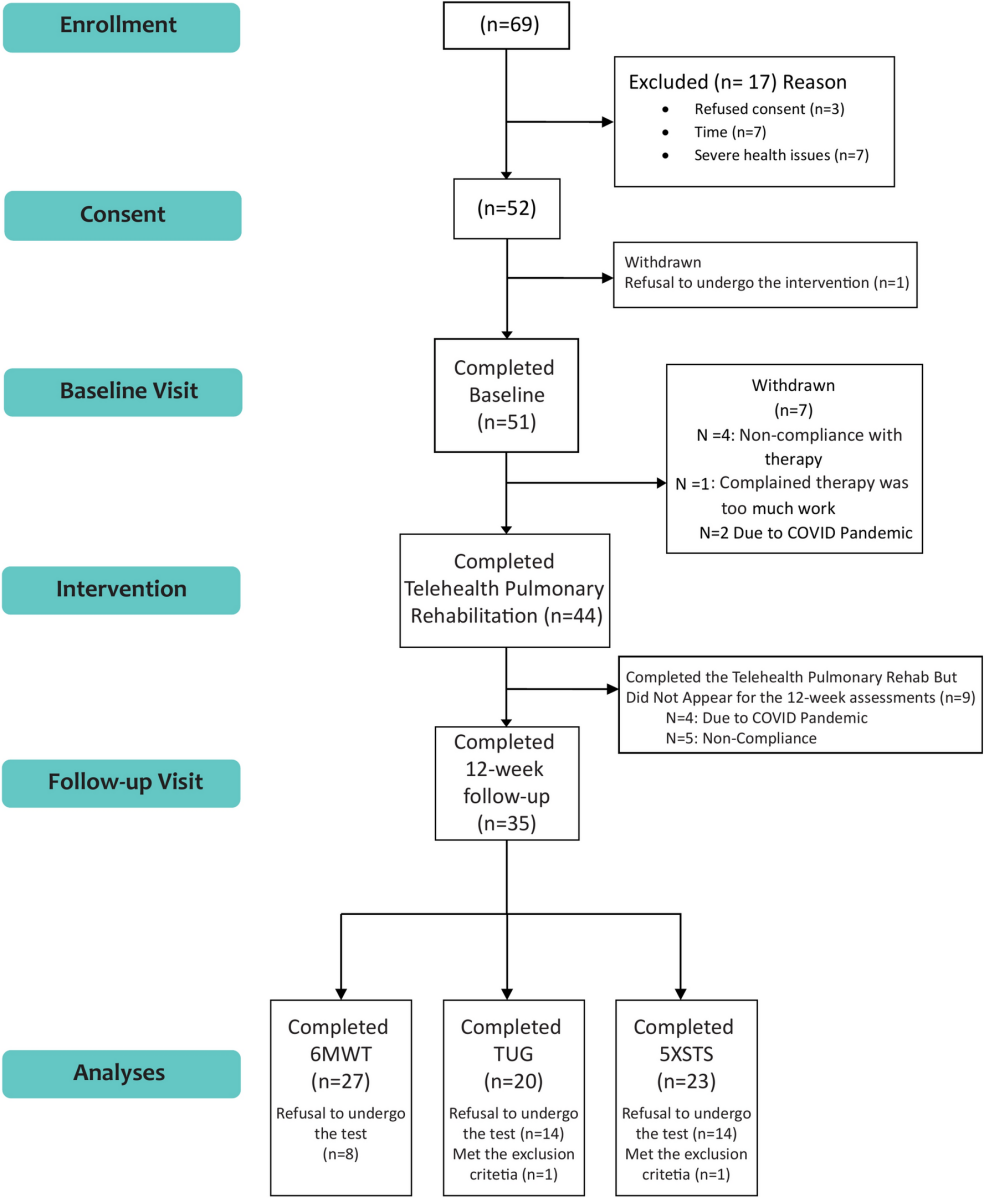
Consolidated Standards of Reporting Trials (CONSORT) diagram.

Figure 2 illustrates the consort chart. Of the 52 participants who gave consent, 51 enrolled in the TPR program. Out of these, 44 successfully completed the program, resulting in an attrition rate of 13.7%. The reasons for participant dropout from the TPR program included refusal or non-compliance (n = 4), feeling overburdened by the program (n = 1), and a mandatory cessation of research activities due to the COVID-19 pandemic (n = 2). When the pandemic-related dropouts are excluded, the attrition rate, reflecting the feasibility of TPR, is calculated at 9.8%. Additionally, 35 participants attended the 12-week follow-up session to undergo outcome assessments and/or agreed to complete the primary outcome measure, with additional 4 participants could not attend the follow-up visit due to mandatory cessation of research activities due to the COVID-19 pandemic. This participation level suggests the feasibility of the primary outcome measure, corresponding to an overall attrition rate of 14%. Detailed patient characteristics for those who completed the 6MWT, 5XSTS, and TUG are outlined in Table 1.

**Table 1:** Patient characteristics at baseline.

Variable	Participants completed 6MWT (n = 27)	Participants completed 5XSTS (n = 23)	Participants completed TUG (n = 20)
Demographics characteristics			
Sex (M/F)	25/2	23/0	20/0
Age (Years)	67 ± 8	69 ± 7	68 ± 9
Weight (kg)	93.82 ± 17.51	92.92 ± 15.36	89.43 ± 16.56
Height (m)	1.74 ± 0.08	1.75 ± 0.06	1.74 ± 0.07
BMI (kg/m^2^)	30.6 ± 5.21	30.28 ± 4.7	29.41 ± 4.89
Clinical characteristics			
Musculoskeletal disease (n)	21	18	16
Cardiovascular disease (n)	15	13	11
Osteoarthritis (n)	17	13	10
Hypertension (n)	22	20	17
Hyperlipidemia (n)	21	17	13
Diabetes (n)	4	3	3
Depression (n)	15	10	8
Smoking history (n)	26	23	19
FEV_1D_ (%)	44 ± 13	43 ± 13	43 ± 13
FVC (%)	66 ± 16	66 ± 15	65 ± 14
FEV_1_/FVC (%)	50 ± 13	47 ± 11	49 ± 13
SPO_2_ (%)	92 ± 3	92 ± 3	92 ± 3

Mean ± standard deviation values. BMI = Body mass index. FEV_1_ = Forced expiratory volume in 1 s. FVC = Forced volume capacity. SPO_2_ = Saturation of Oxygen.

Test scores for the outcomes measured from both the baseline and follow-up visits all showed normal distribution based on the Shapiro–Wilk test (Supplementary 1; Excel File). No adverse event (falls or injuries during the TPR sessions) was reported.

**Fig. 3:**
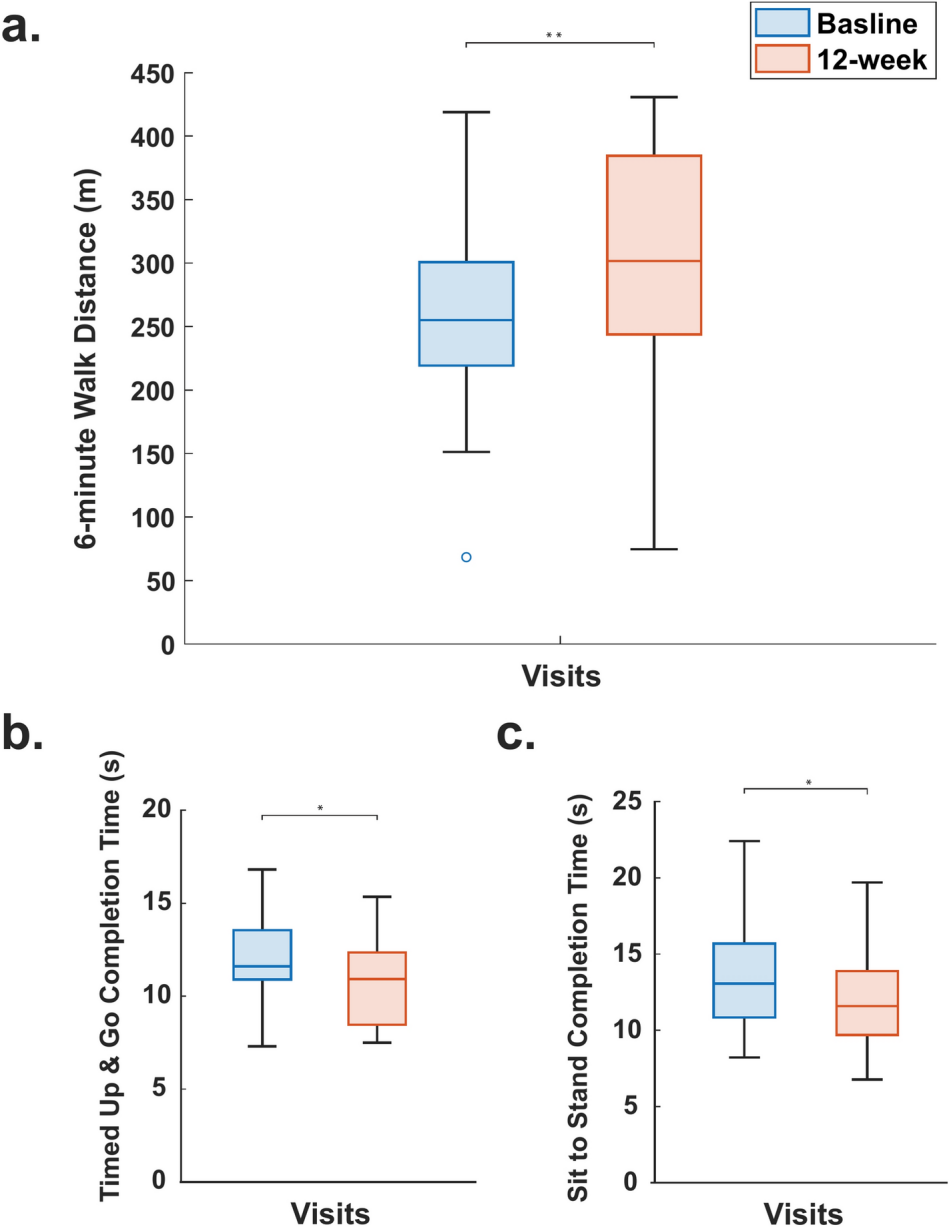
Test score differences between baseline and 12-week follow-up visits during all performed tests. (**A**) 6-min walk distance. (**B**). Timed-up & go completion time. (**C**) Five times sit-to-stand completion time. Findings are displayed in the form of histograms providing the median, the lower and upper quartiles, the minimum and maximum values, and outliers if any are available. *p < 0.05 **p < 0.001.

Following the 12-week TPR program, there were significant improvements in both primary and secondary outcomes (Fig. 3 – Table 2). In summary, at the 12-week mark, there was a 41.3 m (15.7%, *p* < 0.001, Cohen’s d = 0.76) improvement (95% CI 19.8 to 62.7 m) in the 6MWT compared to baseline (Fig. 3A). From the 27 participants that completed the 6MWT, 63% showed an improvement of at least 21 m and up to 120 m, which is considered beyond minimal clinically important difference. Improvements in the TUG and 5XSTS were also observed; TUG showed a mean faster completion time of 1.2 s (95% CI 0.06 to 2.34 s, *p* = 0.019, Fig. 3B), and 5XSTS showed a mean faster completion time of 1.20 s (95% CI 0.03–2.37 s, *p* = 0.022, Fig. 3C). Moreover, Table 2 demonstrate improvements in quality of life, detected through the SGRQ (95% CI 12.18 to 24.17, *p* < 0.001) and CAT (95% CI 9.24–14.97, *p* < 0.001).

**Table 2:** Test score differences between baseline and follow-up visits.

Outcomes	Baseline	12-week	Mean difference		*p*-Value	Cohen’s d
			Unit	%		
Primary						
6-min Walk test (meters)	262.98 (76.03)	304.25 (84.62)	41.27	15.69	< *0.001*	0.761
Secondary						
Timed up & go (seconds)	12.07 (2.17)	10.87 (2.28)	1.20	− 9.94	*0.019*	0.496
5-times sit-to-stand (seconds)	13.36 (3.23)	12.16 (3.12)	1.20	− 8.98	*0.022*	0.445
SGRQ (n.u.)	65.12 (15.74)	46.95 (13)	18.17	− 27.90	< *0.001*	0.813
CAT (n.u.)	28.32 (6.65)	16.21 (6.33)	12.10	− 42.72	< *0.001*	0.906

Mean (Standard deviation). Effect size are presented based on Cohen’s d; small (d = 0.2), medium (d = 0.5), and large (d = 0.8). Significance: α < 0.05. n.u. = no unit. CAT = COPD Assessment Test™. SGRQ = St. George’s Respiratory Questionnaire.

Significant values are in italics.

In comparison between the 35 participants who completed all milestones of the study, including the 12-week follow-up and thus were included in the data analyses (denoted as the completed group), and the remaining 17 participants that consented, including those who may have completed the TPR but did not show up for the 12-week follow-up (denoted as the dropout group); dropouts showed significantly lower weight, had lower proportion of participants with cardiovascular diseases, and had higher saturation of oxygen (Table 3). Additional analyses were completed to compare the 6-min walk distance at baseline between the dropout and completed groups; findings demonstrate a statistical trend with a 35 m higher 6-min walk distance (*p* = 0.196, Cohen’s d = 0.76, 95% CI − 17 to 83 m) performed by the dropout group (302 ± 84 m) compared to the completed group (269 ± 78 m) at baseline.

**Table 3:** Demographics and clinical differences between dropouts and non-dropouts at baseline.

Variable	Participants completed 12-week Follow-up (n = 35)	Dropout participants (n = 17)	*p-*Value
Demographics characteristics			
Sex (M/F)	33/2	15/2	0.589
Age (years)	67 ± 8	70 ± 7	0.282
Weight (kg)	93.17 ± 17.21	80.43 ± 18.85	**0.019**
Height (m)	1.75 ± 0.07	1.73 ± 0.08	0.314
BMI (kg/m^2^)	30.1 ± 5.7	26.9 ± 6.4	0.058
Clinical characteristics			
6-min Walk Test (m)	269 ± 78	302 ± 84	0.196
Musculoskeletal disease (N/%)	27 (77%)	10 (58%)	0.146
Cardiovascular disease (N/%)	22 (62%)	7 (41%)	**0.049**
Osteoarthritis (N/%)	21 (60%)	9 (52%)	0.414
Hypertension (N/%)	28 (80%)	12 (70%)	0.414
Hyperlipidemia (N/%)	26 (74%)	14 (82%)	0.373
Diabetes (N/%)	7 (20%)	5 (29%)	0.891
Depression (N/%)	19 (54%)	8 (47%)	0.136
Smoking history (N/%)	34 (97%)	16 (94%)	0.504
FEV_1D_ (%)	44 ± 14	47 ± 16	0.464
FVC (%)	64 ± 15	63 ± 10	0.836
FEV_1_/FVC (%)	50 ± 13	54 ± 12	0.298
SPO_2_ (%)	92 ± 3	95 ± 1	**0.014**

Mean ± standard deviation values. BMI = Body mass index. FEV_1_ = Forced expiratory volume in 1 s. FVC = Forced volume capacity. SPO_2_ = Saturation of Oxygen. N/% = Number of participants with percentages in brackets.

Significant values are in bold.

## Discussion

The primary objective of this study was to evaluate the effects of a 12-week TPR program, which included both physical and respiratory exercises, on improving functional capacity in veterans with COPD. The primary measure of improvement was the distance covered during the 6MWT. Our findings revealed improvements in 6MWT distance, with an average increase of 41 m after the intervention, surpassing the minimal clinically important difference of 25 m reported in COPD patients^53,54^. These findings are consistent with previous studies. For example, Zanaboni et al. reported an average increase of 40 m in the 6MWT following one year of TPR in patients with COPD^55^. Similarly, Tsai et al. and Oh et al. observed a comparable increase of 40 m in the 6MWT after just 8 weeks of TPR^51,56^.

Lower limb strength and functional mobility, measured using the 5XSTS and TUG tests, respectively, demonstrated statistically significant enhancements in mobility, with a mean reduction of 1.2 s in the TUG test, well within the clinically significant range. (0.9–1.4 s) for COPD patients^57^. The statistically significant improvement in lower limb strength with a mean reduction of 1.2 s in the 5XSTS, nearly reached the minimal clinically important difference of 1.7 s reported in COPD patients.^48^ Both of these results were in line with similar studies assessing pulmonary rehabilitation’s effect on balance^58^ and sit-to-stand test^59^.

Our study not only highlights the effectiveness of pulmonary rehabilitation in improving the functional performance of COPD patients but also emphasizes the potential use of telehealth-rehabilitation as a viable alternative to traditional in-clinic programs. The completion rate of TPR in our study exceeded 86%, with a mere drop-out rate of 9.8%, excluding reasons due to COVID-19 pandemic, a remarkable achievement when compared to previous research and traditional hospital-based rehabilitation programs. Conventional exercise programs suffer from low participation rate (< 50%)^60,61^ and higher drop-out rate compared to home-based rehabilitations^62,63^. For example, Cockram et al. reported 25% attrition rate (58 out of 230 patients with COPD) following 8-week traditional pulmonary rehabilitation intervention^64^. Another study conducted by Singh et al. implemented a 7-week traditional in-person pulmonary rehabilitation program, comprising both exercise and educational components. According to their findings, out of the 267 patients initially enrolled in the program, only 132 successfully completed the entire course of rehabilitation^60^. This completion rate reveals an attrition rate of 50%, which is significantly higher than the 14% observed in the current study. Moreover, as demonstrated in a related review by Sculley et al. with the beginning of COVID-19 pandemic, many healthcare systems providing COPD care had to instantly switch from in-person methods to telehealth methods, and both patients and providers reported satisfaction with telehealth delivery^65^ Telehealth rehabilitation programs were also proven to demonstrate similar effectiveness on improving functional capacity measured by 6MWT as compared to traditional outpatient pulmonary rehabilitation interventions^62,66,67^. It is also noteworthy that benefits of traditional rehabilitation last no more than one year, whereas tele-rehabilitation has more long-standing effects and can be used as a sustainable intervention^33,62^. The notable enhancements we demonstrate in our study likely imply that patients participated in exercise training with a genuine commitment.

Considering the unique challenges faced by veterans, including their old age, increased illness burden, and lower socioeconomic status^68^, this study’s positive outcomes are particularly promising for improving both participation rate and overall quality of life, highlighted by the reduction in overall impact of COPD on health, wellbeing, and daily life demonstrated in our findings through the reduction in overall scores of SGRQ and CAT. In the broader context, veterans’ participation in outpatient appointments within the VA system is limited^69,70^ and accredited pulmonary rehabilitation programs in VHA facilities are restricted and mainly located in urban areas^71,72^, resulting in low in-person participation rate (1.5%) of veterans with COPD in traditional pulmonary rehabilitation programs^73^. Consequently, TPR is an attractive option to overcome these obstacles. The acceptability, safety, and effectiveness of TPR were specifically evaluated among veterans with end-stage pulmonary disease, demonstrating feasibility and the potential for significant effects on dyspnea and exercise capacity^74,75^. The study suggests that exercise enhancement in COPD through 12-weeks TPR program is feasible and may also be effective in preventing deterioration and enhancing physical performance, health status, and overall quality of life. Same results were observed in long-term exercise maintenance of TPR by Zanaboni et al.’s pilot study over the course of two years in ten participants with COPD^55^. Overall, TPR can serve as a potential practical surrogate to hospital-based outpatient maintenance programs^76^. 

Our additional sub-analyses suggest that telehealth pulmonary rehabilitation programs could be more beneficial for COPD veterans that are obese, have higher comorbidities, and deficient oxygen saturation, since dropouts were healthier, which may explain why they chose to opt out. Dropouts covered a higher 6MWT average distance of 35 m compared to participants that completed the study. Such findings highlight the importance of baseline clinical testing as a potential predictor of disease severity to concentrate human resources toward the population in need and ultimately improve their quality of life. Thus, we speculate that one potential reason for dropout from the TPR program could be related to a lower perception of benefit among those who may have higher functional performance. This speculation, however, should be validated in future studies to examine whether baseline functional status can be used as a selection criterion for those who are more likely to benefit from, and complete the TPR program. While this study did not investigate the effects of the TPR program on exercise capacity, hospitalization rates, mortality, and overall wellbeing, systematic reviews indicate that enhancements in measures such as the 6MWT, 5XSTS, and TUG correlate with significant wellbeing outcomes for COPD patients^77–79^. These outcomes encompass aspects like mortality, daily physical activity levels, exercise capacity, and quality of life^77–79^. It is important to note that, on average, COPD patients exhibit a 43% lower level of daily physical activity compared to individuals without COPD^80^ Additionally, our research group has previously demonstrated that patients with COPD experience higher levels of weakness, slowness, and frailty compared to age-matched individuals without COPD^3^. These physical limitations are often compounded by the pulmonary and extra-pulmonary consequences of COPD, leading to altered biomechanics, decreased exercise capacity, increased physical inactivity, decreased exercise tolerance, and heightened frailty. These disparities in functional assessment have also been observed in veterans with COPD^81^. Given these challenges and the significantly low participation rates of veterans with COPD in traditional pulmonary rehabilitation programs, our study emphasizes that tele-rehabilitation could be seamlessly integrated into routine therapy for this population. Furthermore, disease severity has been found to be closely associated with reduced physical activity in this group^82^, underscoring the importance of addressing physical improvements to positively impact respiratory health and overall COPD management.

Although we confirm the effectiveness of TPR in improving functional capacity, it is worth mentioning that despite the 6MWT feasibility (~ 52% completed both visits) which is higher than that reported by the TUG (~ 45% completed both visits) and 5XSTS (~ 39% completed both visits), the 6MWT feasibility is relatively lower than those reported by other studies 51, 55, 56. For instance, Oh reported a 6MWT feasibility of ~ 78% (15/19 participants completed 6MWT on both visits) 56, Tsai et al. reported a 95% feasibility (19/20 participants completed 6MWT on both visits) 51, and Zanaboni et al. reported a 100% feasibility (10/10 participants completed 6MWT on both visits) 55. The higher prevalence of debilitating baseline comorbidities, such as cancer, musculoskeletal, and cardiovascular diseases, among the study population, along with the longer distances Veterans often travel to reach MA hospitals—often over 80 miles—may explain why our study showed lower feasibility rates compared to other studies, as this situation differs notably from that of civilian patients.

Actively recruiting and collecting data during the COVID-19 pandemic is an unforeseen limitation that we had to manage and overcome to maintain research integrity, while making sure patients benefit from the telehealth rehabilitation intervention without being exposed to any additional risks. Although our dataset is rich and provides valuable in-person data before and after the TPR, remote biomechanical data were not collected, which may provide additional beneficial data that could demonstrate ongoing progress to adjust the exercise volume and intensity. Future studies may benefit from remotely measuring biomechanical data to monitor rehabilitation progress and make necessary adjustments. A notable limitation of this study involve the absence of a control group and the predominantly male participants. While this is typical for veteran populations, it may limit the applicability to broader groups. Furthermore, the effects of potential confounding factors were not sufficiently explored, and the modest sample size further constrains the generalizability of our findings. Additionally, the study was limited to veterans living within 80 miles of the Michael E. DeBakey Veterans Affairs Medical Center (MDVAMC) in Houston. This proximity requirement was essential to ensure that participants could attend both the baseline and the 12-week assessments in the clinic, essential for evaluating the study’s outcomes, especially the 6MWDT. However, this exclusion based on travel distance is unusual for TPR programs and could introduce selection bias. This necessitates a cautious approach when interpreting the results and applying them to more diverse populations or settings. Future research should aim to incorporate a more balanced gender representation, control groups, and a more robust examination of confounding variables to enhance the validity and applicability of the findings.

## Conclusion

In conclusion, the results of this study show meaningful improvements in functional capacity and quality of life observed in veterans with COPD after a 12-week TPR program. These improvements, particularly in 6MWT, 5XSTS, TUG, and QoL demonstrate the effectiveness of TPR in enhancing the physical performance and overall well-being of COPD patients. Importantly, this study emphasizes TPR’s promise as a substitute for traditional in-clinic programs, demonstrated by a high completion ratio of 86% and the lack of adverse events in our study. However, this study is limited by several factors, such as the absence of a control group, a relatively small sample size, and insufficient control for potential confounding variables including gender, BMI, age, and other factors that might influence the outcomes. These limitations affect the generalizability of our findings to both veteran and civilian populations. Despite these limitations, the observed meaningful improvement in functional capacity among veterans with COPD, particularly with a medium effect size (Cohen’s d = 0.76), is promising. This finding warrants further exploration through randomized controlled trials.

## Supplementary Information

Supplementary Information.

## Data Availability

Due to limitations set by the Department of Veterans Affairs (VA), there are restrictions on sharing data from veteran studies. Nevertheless, the corresponding author, upon receiving approval from the VA on a case-by-case basis, may be able to share certain data segments deemed appropriate and safe for sharing.
